# Retroviral PBS-segment sequence and structure: Orchestrating early and late replication events

**DOI:** 10.1186/s12977-024-00646-x

**Published:** 2024-06-17

**Authors:** Xiao Heng, Amanda Paz Herrera, Zhenwei Song, Kathleen Boris-Lawrie

**Affiliations:** 1 Department of Biochemistry, University of Missouri, Columbia, MO 65211 USA; 2 Department of Veterinary and Biomedical Sciences, Institute for Molecular Virology, University of Minnesota, Saint Paul, MN 55108 USA

## Abstract

An essential regulatory hub for retroviral replication events, the 5’ untranslated region (UTR) encodes an ensemble of cis-acting replication elements that overlap in a logical manner to carry out divergent RNA activities in cells and in virions. The primer binding site (PBS) and primer activation sequence initiate the reverse transcription process in virions, yet overlap with structural elements that regulate expression of the complex viral proteome. PBS-segment also encompasses the attachment site for Integrase to cut and paste the 3’ long terminal repeat into the host chromosome to form the provirus and purine residues necessary to execute the precise stoichiometry of genome-length transcripts and spliced viral RNAs. Recent genetic mapping, cofactor affinity experiments, NMR and SAXS have elucidated that the HIV-1 PBS-segment folds into a three-way junction structure. The three-way junction structure is recognized by the host’s nuclear RNA helicase A/DHX9 (RHA). RHA tethers host trimethyl guanosine synthase 1 to the Rev/Rev responsive element (RRE)-containing RNAs for m7-guanosine Cap hyper methylation that bolsters virion infectivity significantly. The HIV-1 trimethylated (TMG) Cap licenses specialized translation of virion proteins under conditions that repress translation of the regulatory proteins. Clearly host-adaption and RNA shapeshifting comprise the fundamental basis for PBS-segment orchestrating both reverse transcription of virion RNA and the nuclear modification of m7G-Cap for biphasic translation of the complex viral proteome. These recent observations, which have exposed even greater complexity of retroviral RNA biology than previously established, are the impetus for this article. Basic research to fully comprehend the marriage of PBS-segment structures and host RNA binding proteins that carry out retroviral early and late replication events is likely to expose an immutable virus-specific therapeutic target to attenuate retrovirus proliferation.

**Graphical abstract:**

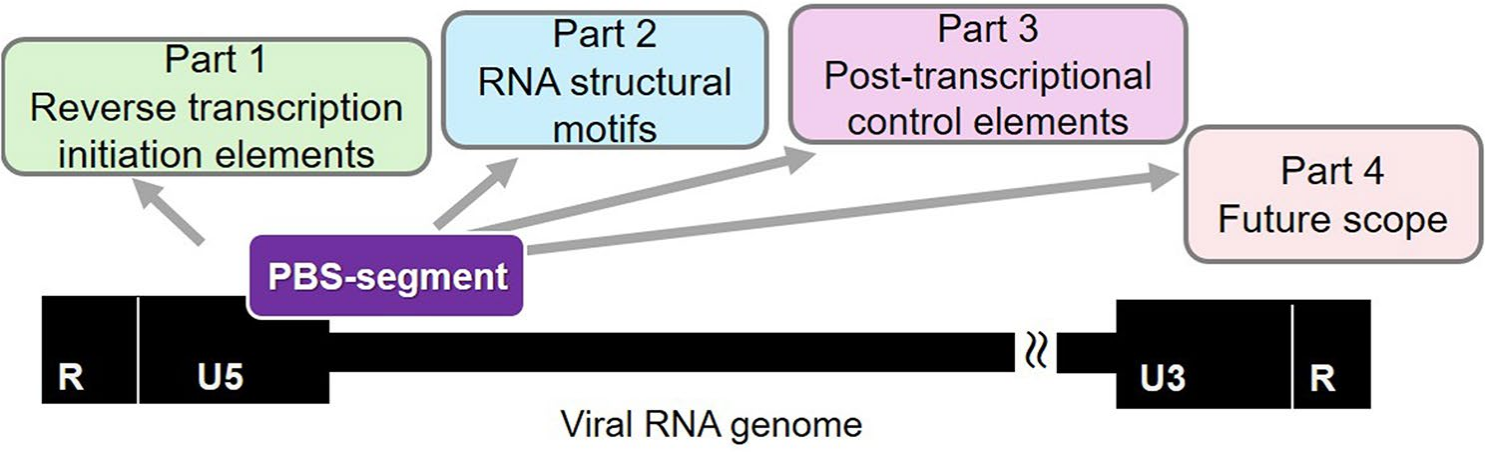

## **Introduction**

**Fig. 1:**
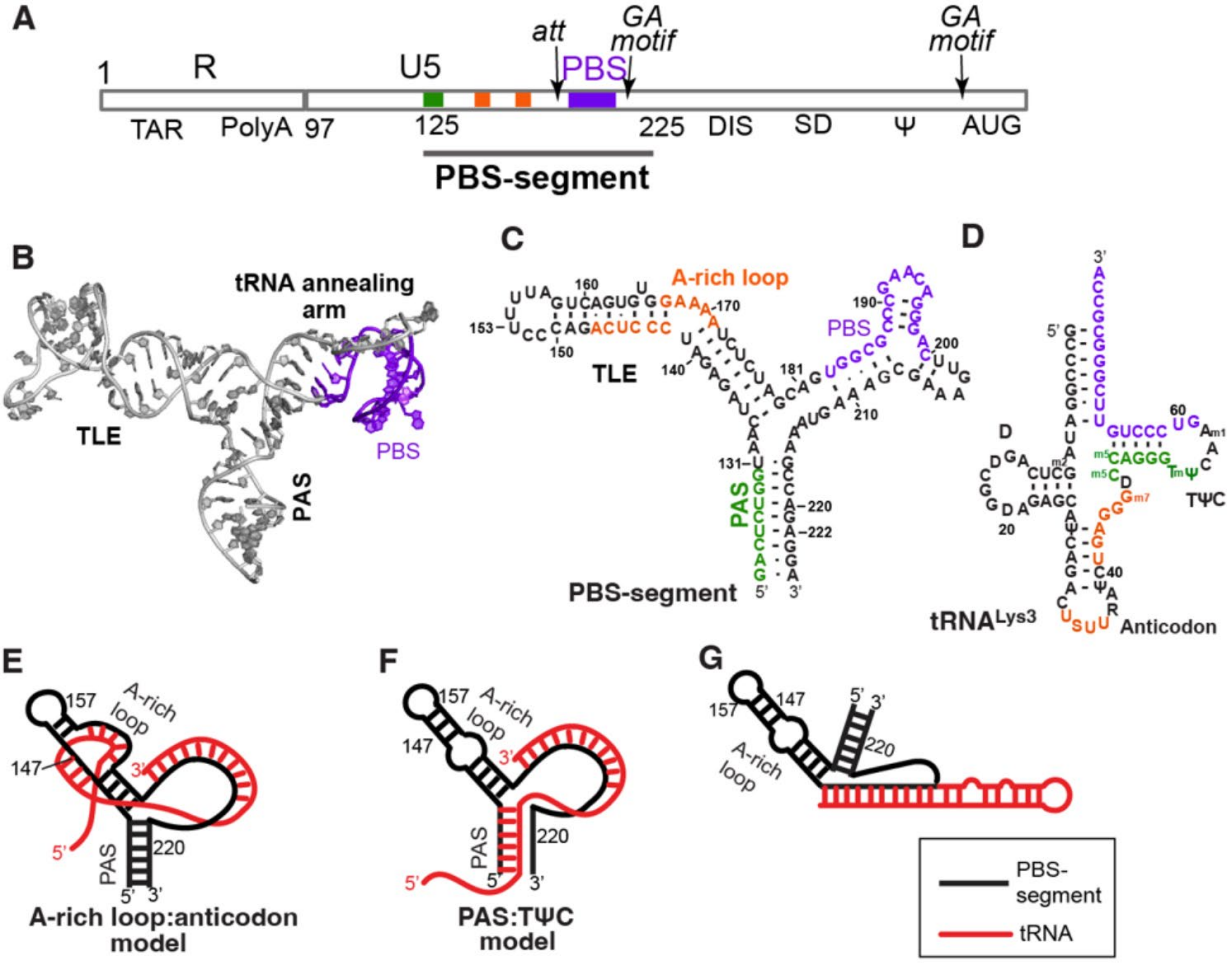
PBS is the primer binding site to initiate reverse transcription. **A**. The context of the HIV-1 5’UTR. Green, primer activation site (PAS); orange, A-rich loop; purple, PBS. **B**. The solution structure of the PBS-segment determined by NMR and SAXS is shown. PDB entry 7LVA. **C**. Secondary structure of the PBS-segment determined by solution NMR and SAXS. Orange, reported sequences involved in activity of the A-rich loop. **D**. tRNA^Lys3^ contains residues complementary to residues in the PBS-segment (S, m5Cytosine). The colors serve to highlight the complementarity between the tRNA residues and their corresponding colored counterparts in A and C. E A-rich loop: anticodon model predicts additional base pairs between the A-rich loop of PBS-segment and the anticodon of tRNA^Lys3^ (red) **F**. PAS: TΨC model predicts additional base pairs between the PAS residues of PBS-segment and TΨC arm of tRNA^Lys3^. **G**. CryoEM results of the reverse transcription initiation complex depict the tRNA^Lys3^ is partially unwound and coaxially stacked on the duplex formed between the PBS and the 3’-22-nt residues of tRNA^Lys3^

The primer binding site (PBS) is positioned downstream of the unique 5’ (U5) region of retroviral RNAs of all retroviruses. PBS is a highly conserved primary sequence motif derived from the 3’-terminus of a cognate tRNA, whose annealing on virion RNA (vRNA) primes reverse transcription initiation of vRNA [1, 2]. The PBS is positioned within a segment of primary sequence designated the PBS-segment (Fig. 1A). Overlapping within the PBS-segment are cis-acting RNA elements necessary for the expression of primary transcripts s made from provirusesproductively infected. Molecular studies have been decrypting an inextricable functional linkage between activities of the PBS-segment in vRNA and viral RNAs assisted by viral proteins and host proteins. The functional repertoire of PBS-segment is immense.

By combining the power of structural biology with functional insights from molecular virology, the HIV-1 PBS-segment was recently elucidated to adopt a thermodynamically stable three-way junction structure (Fig. 1B) [3]. By recognizing the shape of the three-way junction, host nuclear RNA helicase A (RHA/DHX9) is recruited into virions. Results from HIV-1 infected CD4 + T cells demonstrated that the virion-associated RHA promotes the processivity of reverse transcriptase on genomic RNA to bolster infectivity. Such RHA: PBS-segment interaction is also necessary for the epigenetic modification of select HIV-1 m7G-Cap that licenses specialized translation [4]. This article presents a historical view of the PBS-segment structure/function beginning with its classical role for tRNA annealing to prime reverse transcription and then moving to onto host protein interactions within cells for post-transcriptional expression and production of progeny virions.

## Part 1. PBS-segment in early replication events

### PBS is complementary to cognate tRNA

The classical role of the PBS-segment is within vRNA, serving to prime reverse transcription initiation. The cognate tRNA primer is conserved between genera of the *Retroviridae* and within strains of each genus [5]. Every tRNA carries a specific amino acid through isoacceptors that are distinguished by different anticodons. The tRNAs most commonly used as primers for retroviruses are tRNA^Trp^, tRNA^Pro^, tRNA^Lys1,2^, tRNA^Lys3^, tRNA^Met,1^ [1]. For example, vRNA reverse transcription in HIV-1 and other lentiviruses is primed by annealing of the 3´-terminal 18 nucleotides (nts) of human tRNA^Lys3^ onto the complementary PBS residues within the 5’UTR [6] (Fig. 1A). The vRNA of Moloney murine leukemia virus (MMLV) and human T-cell leukemia virus type I (HTLV-I) engage tRNA^Pro^ as the primer, whereas avian leukosis virus (ALV) vRNA reverse transcription is primed by maintained complementarity to tRNA^Trp^ [7].

#### The A-rich loop: anticodon interaction is necessary for tRNA selection and efficient reverse transcription

Observations from different laboratories have provided evidence that PBS and flanking sequences operate together to select the tRNA primer. Even though many tRNA molecules other than tRNA^Lys3^ are co-packaged into HIV-1 virions, transfection of retroviral plasmid containing mutated PBS sequences that complement the 3’ end of a tRNA disparate from the natural primer tRNA^Lys3^ dramatically diminished HIV-1 replication kinetics [8–10]. Similar results were observed in ALV [7], but not in MMLV [11, 12]. These findings exposed the premise that a complexity of factors garners primer usage to carry out the retroviral replication cycle.

When mutations were introduced within the 18 nt PBS of vRNA to be complementary to the 3’- 18-nt terminal residues of non-cognate tRNA (Fig. 1C, purple), replication delays were observed, and after prolonged tissue culture, the mutated PBS reverted to the sequence complementary to tRNA^Lys3^, indicative of strong selective pressure to maintain the native tRNA primer [8, 10]. Molecular virology experiments have provided compelling results that additional interactions between vRNA and its cognate tRNA are necessary for effective virus replication; these include complementarity between A-rich loop of vRNA and anticodon residues within tRNA^Lys3^ (Fig. 1E).

These unpaired A-loop residues (Fig. 1C, orange) are highly conserved in HIV-1 [3], and an analogous bulge is observed in other retroviruses, such as HIV-2 and simian immunodeficiency virus (SIV) that is situated at a comparable distance from the PBS [13, 14]. The intermolecular interaction between the A-rich loop residues of vRNA and anticodon residues of tRNA^Lys3^ is supported by chemical and enzymatic probing studies, as well as solution NMR [5, 15–17]. When mutations were introduced within the 18 nt PBS of vRNA and near the A-rich loop region to be complementary to the anticodon residues of an alternative tRNA, virus replication was observed (Fig. 1C, orange). This strategy was executed to create vRNA complementary to tRNA^His^, tRNA^Met^, tRNA^Gly^, tRNA^Lys1,2^, tRNA^Phe^ and tRNA^Glu^ and studies documented virus replication kinetics were nearly 50% of the wildtype virus [18–25]. Deletion of the A-rich residues negatively impacted virus replication, and the A-rich residues were restored in long-term cultures [26]. From these results, the vRNA sequence complementarity to tRNA anticodon and the extended interaction of tRNA with the upstream A-rich loop motif proved advantageous to maintain the alternative tRNA primer.

Additional insights have been gained from in vitro assays. Marquet and colleagues have established that tRNA priming of reverse transcription proceeds in a two-step manner: (1) the tRNA-specific initiation phase; and (2) the elongation phase [27–30]. Observations of + 3 and + 5 nt extended products during in vitro transcription have suggested the transition between initiation and elongation is rate-limiting [27]. Mutation of the A-rich loop affects both initiation and elongation steps of reverse transcription [27, 31, 32].

Other in vitro assays revealed that the long-range vRNA: tRNA complex requiring the A-rich loop motif diminished affinity for RT, but protected the vRNA from degradation by the RNase H activity of RT in the absence of active reverse transcription [17]. Since RNase H activity of RT can slowly cleave RNA/RNA in the absence of a DNA/RNA hybrid, forming a complex engendered by A-rich loop: anticodon interaction and reducing interaction with RT when reverse transcription is hindered in an environment lacking dNTPs could safeguard the integrity of the vRNA within the virion from the RNase H nuclease activity of RT [17, 33]. Upon the virus entering a host and gaining access to a sufficient amount of dNTPs to support efficient reverse transcription into complementary DNA (cDNA), the RNase H activity directed by cDNA/RNA hybrids would then digest the vRNA [17]. Together, these observations offer a plausible explanation that the adaptation of an alternate tRNA primer requires A-rich loop: anticodon interaction to safeguard the integrity of the vRNA from RNase H activity of RT. There is overlap between the A-rich loop motif and the three-way junction structural motif necessary for post-transcriptional expression of viral transcripts (Part 3) that amplifies positive selection on the PBS-segment.

#### Primer activation site (PAS) in vRNA is identified to base pair with complementary residues in tRNA to promote reverse transcription initiation

Cell-free reverse transcription assays and in vitro biophysical analysis by Berkhout and colleagues identified another HIV-1 vRNA: tRNA interaction that is between the PAS of vRNA (Fig. 1C, green) and the complementary nts within the TΨC arm of tRNA^Lys3^ (Fig. 1F, green). Phylogenetic analysis has revealed the presence of a PAS element in all retroviral genomes [34]. Mutating the PAS sequence reduced the reverse transcription efficiency of vRNA: tRNA complex extracted from virions. Interestingly, mutations in the opposing strand of PAS stem that can release the PAS from base pairing exhibit increased reverse transcription both in vitro and ex vivo [35, 36]. Simultaneous mutation of both 18 nt PBS and PAS to be complementary to tRNA^Lys1,2^ successfully switched primer from tRNA^Lys3^ to tRNA^Lys1,2^ in reverse transcription reactions in vitro when supplied with a pool of tRNAs extracted from calf liver [34]. Although replication was observed of a mutant virus carrying mutations in both PAS and PBS to be adapted to tRNA^Lys1,2^, long-term culturing exposed revertant virus containing a U126C reversion in the PAS. This revertant mutation stabilizes the PAS: anti-PAS interaction by changing the U: G base pair to a C: G base pair [37], implicating positive selection that may be attributable to the post-transcriptional control element (Part 3). These observations support the importance of PAS: anti-PAS interaction in tRNA selection and reverse transcription initiation.

#### The annealing of vRNA with cognate tRNA is facilitated by chaperone protein

The process of tRNA annealing does not occur spontaneously at room temperature as the structures of both tRNA and PBS-segment need to be denatured. The annealing process relies on the chaperone activity of the nucleocapsid (NC) domain of the Gag polyprotein. NC facilitates tRNA annealing by surmounting the energy barrier associated with unwinding thermodynamically stable base pairs in both vRNA and tRNA [38, 39]. The NC domain of Gag is cleaved by viral protease during maturation of virions. The amino acids of NC comprise zinc finger domains and basic residues in the C-terminal tail, which bind and stabilize single stranded RNA residues during RNA unwinding [40–44]. Both the NC domain of Gag polyprotein and the cleaved NC protein have the chaperone activity to promote tRNA annealing, but complete tRNA annealing requires mature NC for optimum priming of reverse transcription [45]. An NMR study uncovered that MMLV NC mediates primer annealing by specifically binding to several regions of PBS-segment and the tRNA^Pro^ primer to expose their complementary sequences for annealing [46], emphasizing the importance of NC in promoting tRNA annealing. In virio SHAPE activities of the HIV-1 vRNA from protease-deficient virions did not show a similar pattern of complete tRNA^Lys3^ annealing as that of the wild type virions [47]. These results posit that the HIV-1 chaperone-assisted annealing is a two-step process initiated by Gag-facilitated annealing at the site of virion assembly, and subsequent re-annealing facilitated by NC in mature virions [48].

Several host factors have been reported to promote tRNA annealing. Inositol phosphate on plasma membrane can stimulate Gag-promoted tRNA annealing, further supporting that the placement of tRNA onto PBS may occur during virus assembly on the plasma membrane [49]. Lysyl-tRNA synthetase (LysRS) interacts with PBS-segment to facilitate annealing of tRNA^Lys3^ onto the PBS-segment. The interaction is mediated by LysRS binding to a tRNA-like element (TLE) in the PBS-segment (Fig. 1C, more in Sect. 1.3a) [50]. SAXS studies suggest that the TLE stem of PBS-segment mimics the anti-codon arm of tRNA^Lys3^, and thus it can bind LysRS to facilitate loading and annealing of tRNA^Lys3^ [51].

Host RHA is specifically recruited into HIV-1 virions [52], and in vitro studies indicate that it cooperates with Gag to facilitate tRNA annealing [53]. Interestingly, host ribosomal protein L7 (RPL7) interacts with the NC domain of Gag and can boost the Gag-chaperoned annealing of tRNA^Lys3^ to PBS-segment in biochemical assays [54, 55]. However, RPL7 in virions has yet to-be assessed, leaving the significance of the biochemical data uncertain.

Annealing can also be accomplished by heating PBS-segment RNA and tRNA mixtures followed by gradually cooling. To assess whether heat- and NC- annealed complex adopt similar structures, various chemical and biophysical experiments were conducted. However, discrepancies of the complex models observed in early studies arose from the different subtypes of HIV-1 lab-adapted sequences. The recombinant circulating subtype A strain, HIV-1 MAL features a 23-nt insertion downstream of PBS relative to the subtype B lab strains (NL4.3, HXB2). In the case of HIV-1 MAL PBS, both the heat- and NC-annealed complex formed between a 124-nt PBS-segment RNA (nt 110–233) and tRNA^Lys3^ and the A-rich loop: anticodon interaction was supported by enzymatic probing and NMR studies [16, 17]. The NMR and FRET studies of a 77-nt MAL vRNA (nt 120–196) heat annealed with tRNA^Lys3^ support the formation of PAS: anti-PAS interaction [56]. In the case of HXB2 (subtype B), dynamic PAS: anti-PAS interaction was observed in NC-annealed vRNA: tRNA complex [57]. NL4.3 sequence (subtype B) A-rich loop: anti-codon interaction was observed only in the NC-annealed complex but not the heat-annealed complex [17]. The different results observed in various studies involving different vRNA sequences and annealing methods highlight the challenges associated with studying the tRNA annealing process in vitro.

### Assembly of reverse transcription initiation complex (RTIC)

Recent high-resolution cryoEM studies of the HIV-1 RTIC have revealed molecular interactions between HIV-1 RT, PBS-segment and tRNA^Lys3^ [58]. To counter the rapid disassociation of RT from the vRNA-tRNA complex, the vRNA-tRNA was cross-linked to an RT containing a Q258C mutation in the p66 unit, creating a disulfide bond with the N2-cystamine-dG at position 71 of the tRNA^Lys3^. The structure of RTIC revealed a continuous RNA helix spanning the RT binding cleft, suggesting that the tRNA had refolded and adapted an extended helical conformation in the complex (Fig. 1G) [58]. In this study, the tRNA^Lys3^ and PBS-segment were heat annealed. Consistent with previous findings [17], the heat-annealed complex between PBS-segment and tRNA^Lys3^ did not form extended interactions other than the 18-nt base pairings. Modeling of the RNA structure in the RTIC within the 8 Å cryoEM map proposed that stacking of the double-stranded PBS helix formed between tRNA^Lys3^ and the 18-nt PBS on the 5’ end of tRNA^Lys3^ within in the cleft of RT (Fig. 1G). The structure model shows that HIV-1 PBS-segment structure re-arranges when annealed with tRNA^Lys3^ in conformation to serve as the template to initiate reverse transcription.

### Host factors are recruited by PBS-segment to facilitate reverse transcription

#### LysRS delivers cognate tRNA^Lys3^ to PBS for primer annealing

tRNA^Lys3^ is incorporated into virions by host LysRS via interaction with viral Gag and Gag-Pol proteins [48, 59–64]. Explorations into the mechanism by which LysRS delivers tRNA^Lys3^ to PBS for annealing reveal that LysRS specifically binds to the TLE of the PBS-segment. The specificity stems from the molecular mimicry between the TLE stem loop and the anticodon of tRNA^Lys3^, both featuring a U-rich loop. Mutation of the U-rich sequences in TLE reduced the affinity for LysRS, and subsequently reduced the amount of annealed tRNA^Lys3^ in virions [50, 51].

#### Host nuclear RHA is incorporated into virions and serves as an RT processivity co-factor

The nuclear RHA is a component of infectious HIV-1 virions to bolster virion infectivity acting at several steps [52]. It coordinates with Gag during tRNA annealing, amplifying both the abundance of tRNA^Lys3^ on vRNA and the ability of tRNA^Lys3^ to prime the initiation of reverse transcription [53]. In primer extension studies recombinant RHA significantly improves reverse transcription efficiency without affecting the kinetics of single nt incorporation of RT. It unwinds RNA from the 3’- to 5’- direction, potentially resolving roadblocks such as RNA secondary structure and possibly NC proteins on the RNA template during reverse transcription [65]. Therefore, RHA functions as an RT processivity co-factor during reverse transcription. Unlike other viruses such as Influenza virus and SARS-CoV2, HIV-1 does not encode its own viral helicase. Instead, it captures RHA using the PBS-segment as the bait to tether this abundant host factor to accelerate reverse transcription, leading to the synthesis of double-stranded proviral DNA. RHA is tethered to the PBS-segment of virion precursor RNA and incorporated into virions during genome packaging [65, 66].

RHA is a member of the DEAD box superfamily of host proteins, has two N-terminal double-stranded RNA binding domains of the αβββα family (dsRBD1 and dsRBD2). Both dsRBD1 and dsRBD2 are necessary for cognate RNA binding [67]. NMR and other biophysical studies identified that dsRBD1 specifically binds to the three-way junction structure of PBS-segment in the HIV-1 5’UTR (Fig. 1B). The structural attribute required for RHA affinity is the angle between the TLE and PAS stem. This specific interaction encourages additional hydrogen (H)-bonds between dsRBD1 and PBS-segment RNA, as compared to low affinity dsRBD1 binding to a straight hairpin structure [3]. These H-bonds clamp RHA onto PBS-segment to aid virion incorporation and prevent RHA sliding on RNA via its helicase activity.

### Controversial issues of tRNA annealing and genome packaging

The annealing of tRNA onto vRNA primes reverse transcription. Controversy reins as to whether tRNA annealing onto PBS-segment occurs prior, during, or after the vRNA packaging step of virion assembly, and if the mechanism varies among different retroviruses. The spatial and temporal regulation of tRNA annealing has been investigated by various approaches, such as comparing the relative concentrations of tRNA in cytosol and virions.

HIV-1 specifically recruits tRNA^Lys3^ into virions by tRNA^Lys3^-bound LysRS [60, 63, 64], resulting in ~ 10 fold enrichment of tRNA^Lys3^ relative to cytosolic levels [62]. Unlike HIV-1, MLV does not selectively enrich tRNA^Pro^ in virions [68]. NC binding to the high affinity sites in tRNA^Pro^ and the U5-PBS segment in the MLV 5’UTR exposes the complementary sequences on both RNAs to promote annealing [46]. This scenario postulates tRNA annealing can occur in an environment where the local concentration of vRNA and tRNA is low, for instance cytosol. By contrast, NC interaction with HIV-1 PBS-segment is relatively weak and non-specific. Hence it is plausible that tRNA annealing onto HIV-1 RNA takes place in an environment enriched with tRNA^Lys3^, such as within virions. Indeed, the two-step annealing model supported by the in virio SHAPE probing experiments is in line with the hypothesis that tRNA^Lys3^ annealing occurs first in cytosol by Gag and then by NC in virions to form a mature annealed complex [47]. Additionally, inositol phosphate, a component of plasma membrane, can stimulate Gag-promoted tRNA annealing, providing correlation that the placement of tRNA^Lys3^ onto HIV-1 PBS proceeds as virions assemble on the plasma membrane [49].

Retroviruses package two copies of vRNA into virions, which in HIV-1 is mediated by Gag preferentially binding to the dimeric 5’UTR. Annealing of tRNA^Lys3^ or an 18-nt DNA oligonucleotide complementary to PBS promotes intermolecular dimerization of the 5’UTR [69]. This observation implies that primer annealing is likely to shift the RNA structural equilibrium towards the dimer conformation favored for genome packaging. FRET studies showed that tRNA^Lys3^ annealing affected 5’UTR structural dynamics by promoting the kissing-dimer, which is believed to be an intermediate state of vRNA in the transition into an extended dimer in virions [70]. However, when PBS-segment was deleted, genome packaging efficiency in transfected cells was unaffected, suggesting the PBS-segment and thereby tRNA annealing was dispensable to direct genome packaging [71–74]. The controversies are likely to derive from the discrepancies between in vitro and in vivo systems.

## Part 2. Structural perspectives of PBS-segment through the prism of molecular genetics

### PBS-segment likely adopts different conformations in the dynamic 5’UTR structures

**Fig. 2:**
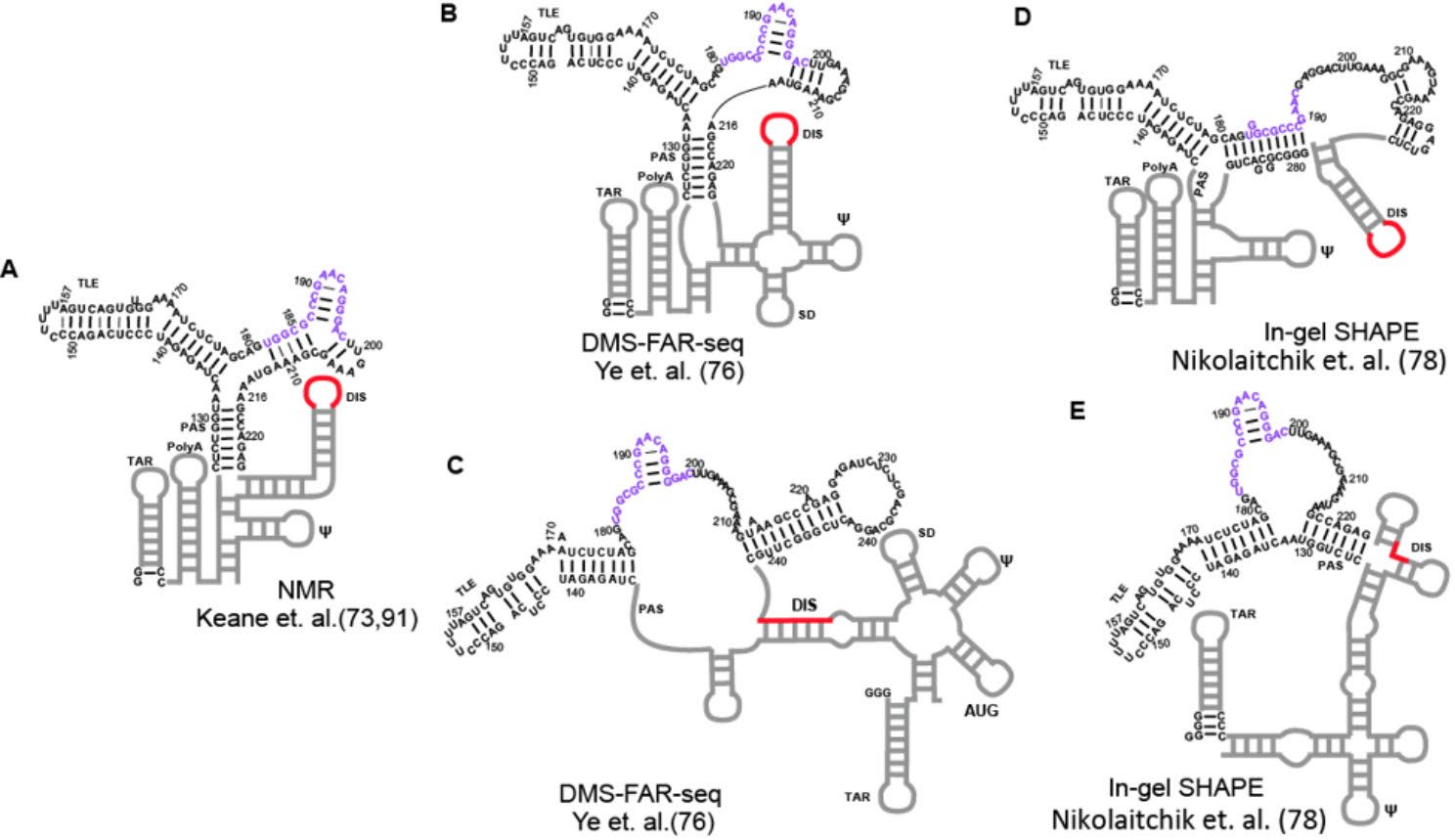
Secondary structure of the PBS-segment modeled by NMR and chemical probing taking into account potential transcription start site heterogeneity. **A**. The secondary structure model of the dimeric 5’UTR was determined by NMR [74, 93]. **B**-**C**. The secondary structure model of 5’UTR starting with GG and GGG, respectively (**B** and **C**) based on FARS-seq (79). D-E. The secondary structure model of 5’UTR starting with GG and GGGG, respectively (**D** and **E**) based on in-gel SHAPE [79]. Purple, tRNA annealing sequence; Red, dimer-promoting sequences, DIS

Phylogenetic studies of the HIV-1 sequences in the Los Alamos HIV-1 database revealed that ~ 22% of HIV-1 sequences contain a 23-nt insertion downstream of the tRNA annealing site; these variants belong to subtype A and circulating recombinant forms. Different structural models have been proposed for the PBS-segment without and with the 23-nt insertion. Phylogenetic analysis of the HIV-1 PBS-segment sequences without the 23-nt insertion supported a conserved three-way junction structure [3]. In addition to the 18 residues that are complementary to the 3’- end of tRNA^Lys3^ and nearly 100% conserved, the bottom stem of TLE hairpin is highly conserved and covariations to maintain tRNA base pairing are observed. Mutational scanning that predicts the impact of mutations at each nt position on PBS-segment structure posited that the formation of the TLE bottom stem is necessary to maintain the overall three-way junction structure of the PBS-segment [3]. Indeed, the TLE hairpin was predicted in most of the PBS-segment structural models from a variety of experimental approaches (Fig. 2) [50, 51, 75–79].

Since the 5’UTR can fold into monomeric and dimeric structures (82,83), PBS-segment and cognate proteins may contribute to the equilibrium of monomer: dimer 5’UTR conformations by shifting local base pairings. Given the fact that both HIV-1 vRNA and the genome-length gag mRNA are the same unspliced primary sequence, there has been considerable research interest in understanding how the fate of these unspliced RNAs is determined by the 5’UTR monomeric or dimeric structures.

Early studies of 5’UTR structures investigated HIV-1 RNA capacity for dimerization and genome packaging efficiency. The Berkhout group predicted that the gag AUG can form long-range base pairing interaction with residues in U5 that occluded AUG from translation initiation. The LDI-BMH model (LDI = long distance interaction; BMH = branched multiple-hairpin) proposed the U5: AUG pairings facilitated the BMH conformation, which exposed DIS to promote dimerization of virion precursor RNAs to be packaged into virions as vRNA [80]. This model postulated the BMH confirmation of the 5’UTR supports RNA dimerization and packaging into virions whereas the LDI model with AUG forming a local hairpin supports translation. The U5: AUG long range interaction in the dimer conformation was confirmed by NMR and *in virio* SHAPE probing [74–76, 81]. However, the 5’UTR mutants favoring the LDI conformation and inhibiting dimerization failed to promote translation [82]. NMR studies of the 5’UTR successfully detected the U5: AUG long-range interaction in the dimer conformation, and that DIS is sequestered by pairing with residues in U5 [83]. The structure of the PBS-segment was predicted to be the same in the LDI and BMH conformers [80] and provides an explanation for the observation that favoring the LDI conformation and inhibiting dimerization failed to promote translation [82]. Important translation regulatory information lays within the structure of the PBS-segment [3, 4, 84] (Part 3).

HIV^NL4−3^ molecular clones containing 5’UTR substitutions based on the NMR structural studies were constructed to individually stabilize the dimer-prone or monomer conformations. Then, for the first time, the translation rates of the viral mRNAs were measured in CD4 + T cells, building on the results from study of HIV translation in reporter systems or subgenomic vector RNAs [85]. Statistical significant differences in Gag translation rate were not observed by stabilizing monomer or dimer conformers; the steady state rate of HIV-1 Gag protein synthesis remained similar. However, the viral mRNA translation rate was significantly increased by destabilization of the PolyA stem, which perturbs the PBS-segment. Intricate measurements of ribosome loading by sensitive quantitative PCR validated the conclusion that the key regulatory parameter is not monomer-dimer equilibrium, but the structural perturbation that influences host factor interaction with PBS-segment (Part 3).

### Heterogeneous HIV-1 5’UTR originates during provirus transcription and determines RNA fates

The retroviral transcription start site (TSS, + 1) corresponds to the junction of U3 and R in the provirus 5’ long terminal repeat (LTR). HIV-1 sequence at U3-R contains three guanosines. In the mid-1980’s, polyadenylated RNA from H9 /HTLV-III cells was analyzed by sensitive S1 nuclease protection assays and primer extension assays with radioisotope. The radiolabeled products of S1 nuclease digestion identified the majority of transcripts were attributed to m7G-Cap and guanosine dinucleotide [86]. Though barely detectable, the assays revealed one-nt species above and below the majority band that conceivably were attributable to TSS heterogeneity. Results of primer extension with the radiolabeled primer identified 5’ residues were GG and GGG [87]. Both the S1 probe and primer for extension were positioned upstream of the 5’ splice site, so the assay results were inclusive of the 3 subsets of viral transcripts (unspliced 9 kb, incompletely spliced, 4 kb; and fully spliced RNA isoforms, 2 kb).

The initial characterizations of HIV-1 RNA structure identified TSS heterogeneity, and recently the issue reemerged in studies that employed reverse transcriptase for 5’ rapid amplification of cDNA ends (5’RACE), linker ligation and sequence analysis. Recent studies identified TSS heterogeneity influences the fate of viral transcripts [88]. Synthetic 5’G RNA support reverse transcription as measured by successful first strand transfer, whereas 5’-GG and 5’-GGG RNA did not [88]. Another study by S1 nuclease protection and 5’ RACE and sequence analysis identified HIV-1 5’-GG and 5’-GGG RNAs predominant in polysomes [88, 89], indicating functionality for viral protein translation. Genomic RNA enriched in virions exhibit the 5’-G TSS.

### HIV-1 5’UTR conformation can be perturbed by TSS heterogeneity

Covalent linkage of methyl-7G-Cap (m7G-Cap) to TSS is a co-transcriptional process catalyzed by enzymes tethered to the C-terminal domain of RNA polymerase II (CTD) [90, 91]. The m7G-Cap is sequestered by nuclear cap-binding complex (heterodimeric CBP80-CBP20, CBC) as a quality control checkpoint prior to transcriptional elongation, intron splicing, and deposition of nuclear export factors. The methyl group on the guanosine cap does not affect the Watson-Crick edge of guanosine and thus the m7G-Cap can base pair with a cytidine. How the TSS heterogeneity and m7G-Cap modification affect the structure of the 5’ UTR has been investigated in NMR studies with synthetic RNAs.

Data collected by the Summers group identified that the m7G-Cap and the number of adjacent G residues significantly affect the conformation of the 5’-UTR in-solution. Their RNA structural studies took into consideration that the m7G-Cap is characteristic of transcripts synthesized by RNA polymerase II. Synthetic HIV-1 RNA of 356 nt and beginning with m7G-Cap and three guanosine residues (5’-GGG) folds into a monomeric structure. They concluded that the m7G-Cap was unpaired for interaction with m7G-Cap-binding proteins and indeed identified interaction with cap binding protein eIF4E and that the m7G-Cap can be decapped by hDcp2 [92]. They identified that the 5’-GGG engaged in Watson-Crick base pairing with cytosine residues at C57-C58-C59. The conformation engendered by m7G-Cap-GGG influenced the structural conformation of the distal 5’UTR in which DIS base pairs with U5 to sequester the dimer promoting sequence. This may potentially explain the monomeric m7G-Cap-GGG RNA is not efficiently packaged as vRNA [92].

By comparison, synthetic RNA beginning with m7G-Cap and one guanosine preferentially folds into a dimer conformation where AUG and SD (splicing donor) are occluded. They are sequestered by complementary base pair interaction whereas DIS is exposed [92]. The 5’ terminal m7G-Cap-G base paired with C57-C58, and thus the m7G-Cap was not readily bound by eIF4E or decapped by hDcp2 [92]. These results demonstrate that m7G-Cap and TSS heterogeneity influence the 5’UTR structural equilibrium between monomer and dimer conformations in-solution.

Since the m7G-Cap can base pair with cytosine and contribute to RNA secondary structure in a way similar as a guanosine, other studies have used guanosine in synthetic 5’-GG and 5’-GGGG transcripts to recapitulate Cap-G and Cap-GGG RNA folding, respectively [77, 79, 89]. It is worth noting that the NMR studies of the 5’UTR with m7G-Cap were carried out with the 5’UTR sequence of the MAL strain, which contains 23-nt insertion in the PBS-segment. MAL represents 20% of HIV-1 strains circulating in patient populations. Notably, despite the extra nts, the upstream base paired regions of the dimeric m7G-Cap-G 5’UTR are similar as the previously determined dimeric 5’UTR structure with 5’-GG, in which the PBS-segment adopts a three-way junction structure without the 23-nt insertion (Fig. 2A) [74, 93]. The structural differences between the monomeric 5’-GGGG and dimeric 5’-GG 5’UTR structures were ascertained in functional analysis of RNA structure-sequencing (FARS-seq) [77], which is a SHAPE-based, model-free method that enables identification of RNA helix structures. This is achieved by introducing mutations to one strand of RNA for disrupting base pairing, and subsequently observing increased SHAPE reactivity in the other strand of RNA [77]. The FARS-seq studies reveal that the secondary structure of PBS-segment is different between 5’-GG dimer and 5’-GGG monomer 5’UTR (Fig. 2B and C) [77]. Notably, while the PBS-segment structure in the dimer 5’UTR (Fig. 2B) agrees with the three-way junction solution structure (Fig. 1B), the PAS stem no longer exists in the monomer 5’UTR (Fig. 2C).

A separate SHAPE study of 5’-GGGG and 5’-GG 5’UTR RNA to recapitulate Cap-GGG and Cap-G RNAs, respectively, reveals different secondary structural patterns (Fig. 2D and E) [79]. Interestingly, the three-way junction-like structure of the PBS-segment with PAS stem and TLE stem is formed in the monomeric 5’-GGGG RNA (Fig. 2E), but not in the dimeric 5’-GG RNA (Fig. 2D). In the dimeric 5’GG RNA model, the tRNA annealing site base pairs with the linker connecting DIS and 5’ splice site (Fig. 2D).

While the exact pairings vary between these folding models of the PBS-segment, in these studies the most prominent structural variations in the PBS-segment are evident between the monomeric and dimer-prone 5’UTRs (Fig. 2). In line with these findings, SHAPE analysis of the feline immunodeficiency virus (FIV) 5’UTR recapitulated that PBS-segment structure differs between the monomeric and dimeric 5’UTRs [94]. The structural variations centered on the PBS-segment in these models posit that its structural perturbation holds the potential to inhibit various viral replication events. A thorough exploration of the dynamic structural isoforms of PBS-segment in these viral transcripts will garner valuable insight into the newly discovered roles of the PBS-segment in post-transcriptional control of viral RNA. Post-transcriptional regulation mediated by the PBS-segment is necessary and critical for the balanced expression of many gene products from a single primary transcript, the epigenetic modification of select transcripts by host enzymes and the biphasic translation that switches between translation initiation mechanisms (Part 3).

## Part 3. PBS-segment sequence and structure: roles in late phase viral replication

### PBS-segment contributes to the balanced expression of retroviral intron-containing and fully spliced mRNAs

**Fig. 3:**
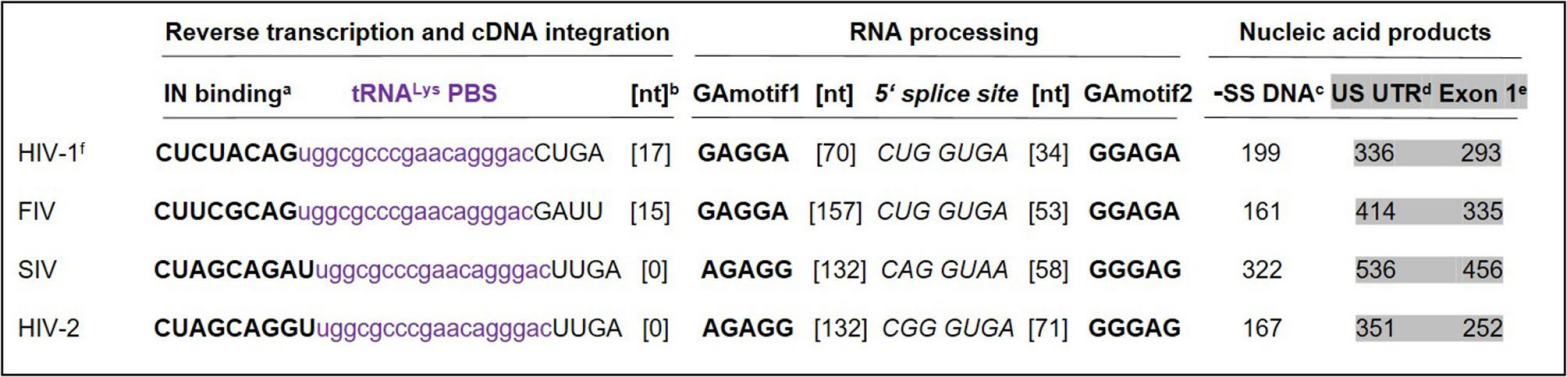
Representative lentiviruses exhibit similar configuration of PBS and flanking sequence motifs. Integrase (IN) binding motif fosters cDNA integration to form provirus. Two GAmotifs flank the 5’ splice site that facilitate balanced expression of transcripts that are fully spliced, incompletely spliced and unspliced (US). The initial nucleic acid product of reverse transcription is minus strand strong stop DNA (-SS) that corresponds to the RU5 region of the 3’ long terminal repeat. The products of host transcription and RNA processing are the US and spliced RNAs with UTR length from 293 to 536 nt. ^a^IN binding site; ^b^[number of nts between indicated motifs]; ^c^Minus strand strong stop DNA); ^d^Approximate number of nt between the transcription start site and the gag AUG; ^e^Approximate number of nt between the transcription start site and the 5’ splice site ;^f^Retrovirus sequence identifiers. HIV-1: NC_001802; Feline immunodeficiency virus: M25381.1; Simian (macaque) immunodeficiency virus, isolate 239: M33262.1; Human immunodeficiency virus 2: NC_001722.1

The configuration of PBS with flanking sequence motifs is highly similar among retroviruses. As summarized in Fig. 3, PBS of representative lentiviruses is complementary to tRNA^Lys^ (purple). This tRNA primes reverse transcription to generate the minus strand strong stop DNA (-sssDNA). The -sssDNA corresponds to the R and U5 regions of the long terminal repeat (LTR). The -SS is inclusive of the Integrase (IN) binding site, also known as attachment (*att*) site (Fig. 3, bold). IN binding this *att* site is critical for cutting and pasting cDNA into the host chromosome to form the 3’ LTR of the provirus.

The provirus is transcribed by RNA polymerase II into the primary transcript that is processed into viral alternatively spliced mRNAs and intron-containing RNAs that encode regulatory, accessory and virion structural proteins. Precisely balanced stoichiometry between the viral spliced and unspliced transcripts is required for efficient viral replication. Recently identified, two guanosine-adenosine motifs (GAmotif1 and GAmotif2) flanking the 5’splice site are required for the balanced expression of the intron-containing viral RNAs [95]. Conservation of the GAmotifs between genetically simple and more complex retroviruses was documented, which posits a common mechanism within the *Retroviridae*.

Results of GAmotif mutagenesis, RNA-directed proteomics, RNA electrophoretic mobility assays and siRNA downregulation studies identified SFPQ/PSF interacts with the GA-motifs for the natural stoichiometry of the viral transcripts [95, 96]. SFPQ/PSF is conserved amongst the hosts of retroviruses. Further investigation is warranted to identify the molecular mechanism of the GAmotifs and SFPQ/PSF in modulating the hallmark stoichiometry of retroviral RNAs.

#### HIV-1 hyper methylated Cap significantly bolsters the proliferation and infectivity of HIV-1

Over a decade ago, the Jeang laboratory published the surprising finding that the m7G-Cap of HIV-1 Rev/RRE-dependent mRNAs is a substrate for host trimethyl guanosine synthase 1 (TGS1) [97]. The well-known substrates of TGS1 had been limited to the noncoding small nuclear RNAs and small nucleolar RNAs that facilitate assembly and maturation of spliceosome and ribosome ribonucleoproteins (RNPs), and yeast telomerase RNPs [98, 99]. TGS1 is an m7G-Cap binding protein that catalyzes the addition of two methyl groups at the N2-position of the m7G-Cap to form m2, 2, 7 G-Cap (trimethyl guanosine, TMG-Cap) [100]. TGS1 engages m7G-Cap by an aromatic sandwich motif that is conserved in CBP20/NCBP2, DCP2 and eIF4E. TGS1 is released from the TMG-Cap in a process involving CRM1 [100]. Evidence was provided that TGS1 promotes Gag production from Rev/RRE-dependent mRNAs in cultured cells and CD4 + primary cells [97, 101]. Recently shown, virion production from TGS1-deficient cells diminished to 10% and the virions were poorly infectious. Virion infectivity on CD4 + T cells diminished by a factor of 1000 compared to normalized control virions [4].

Since the discovery of TMG-HIV-1 RNA, host TMG-mRNAs have been unveiled. First to be identified were selenoprotein transcripts (e.g. Methionine Sulfoxide Reductase B1/SelR), which protect cells from oxidative stress [101]. Next, host TMG-mRNAs were identified and shown to control neoplastic growth: the junD AP1 transcription factor subunit; dhx9 that encodes RHA; and the tgs1 mRNA [102, 103]. RHA is a critical RNP component for TGS1 recruitment to mRNAs for cap hyper methylation [4]. Downregulation of RHA or TGS1 by RNA interference abolishes TMG-Cap from HIV-1 RNA, junD, dhx9 and tgs1 mRNA [4, 102, 103]. Together these studies have revealed that TMG-Cap licenses HIV-1 Rev/RRE-dependent RNAs, and host junD, dhx9 and tgs1 mRNAs for specialized translation independently of eIF4E, and established a new paradigm for host control of mRNA translation.

#### HIV-1 m7G-multiply spliced mRNAs and TMG-Rev/RRE-dependent mRNAs are substrates for biphasic translation

For decades, controversy has persisted as to the molecular basis for HIV-1 translation despite physiological downregulation of eIF4E. eIF4E activity is promptly repressed by hypo phosphorylated 4EBP [100]. 4EBP is the allosteric repressor of eIF4E and target of the kinase, mTOR. mTOR hyper phosphorylates 4EBP during cell proliferation, but its activity is downregulated by oxidative stress, diminished nutrient signaling and normal cell cycle transition from G1/S to G2/M. HIV-1 Vpr upregulates hypo phosphorylated 4EBP in a manner that mimics mTOR downregulation, unveiling antagonism between HIV-1 and eIF4E activity [104].

**Fig. 4:**
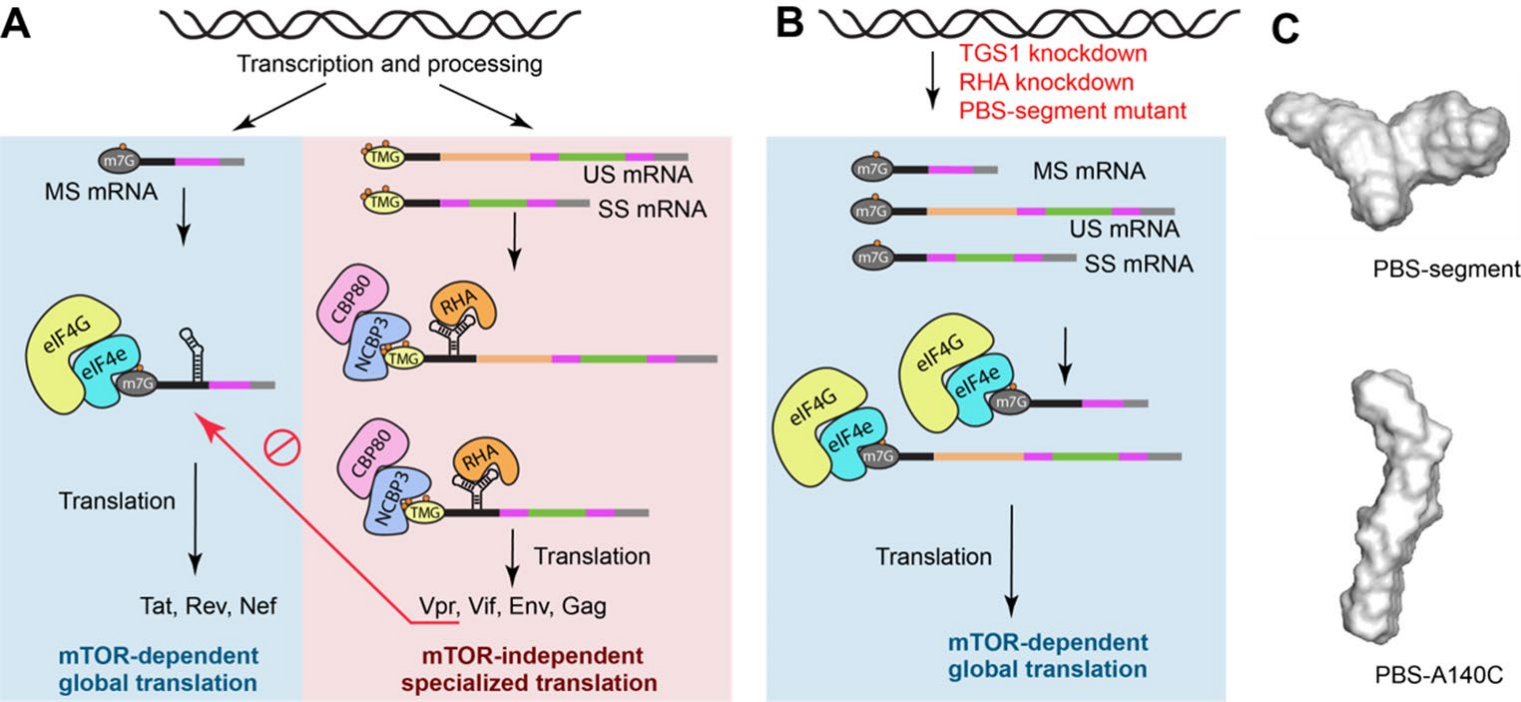
PBS-segment is necessary for m7-G-Cap epigenetic modification and biphasic translation. Completely processed HIV-1 transcripts exhibit m7G-Cap and undergo canonical CBC exchange to eIF4E. Tat, Rev, Nef mRNA translation is promptly attenuated by eIF4E inactivation by hypo phosphorylated 4EBPs. The m7G-Cap of HIV-1 Rev/RRE-dependent intron-containing transcripts are subject to hyper methylation by TGS1 in a process that requires PBS-segment and host RHA/DHX9. TMG-Cap license specialized translation that is unaffected by the eIF4E inactivation. **B**. Downregulation of TGS1 or RHA, or introduced mutation on the PBS-segment abolished TMG-Cap on HIV-1 mRNAs and led to mTOR-dependent translation of virion proteins. **C**. Ab initio model of PBS-segment (top) and PBS-A140C (bottom) derived from SAXS data are shown

Whereas rapamycin is a nonspecific inhibitor of mTOR, catalytic site-selective small molecule inhibitors of mTOR have been developed. Low dosage Torin-1 (50 nM) in CD4 + T cells has been shown to have little effect on host or HIV-1 RNA steady state and validated cell viability maintains during short term treatments (e.g. 20 h). To measure the effect of downregulation of eIF4E-activity on HIV-1 translation, cells were treated with low dose Torin-1 for 20 h and metabolic labeling was carried out for 30 min. Results identified robust de novo synthesis of Gag and Env proteins (gp160/gp120), but attenuated synthesis of Tat, Rev, Nef and host GAPDH [4]. Moreover, virus growth persists in primary CD4^+^ T cells, MT-4 and CEM×174 CD4 + lymphocytes. After 4 days, lack of HIV-1 regulatory protein synthesis results in downregulation of viral intron-containing RNAs and reciprocal upregulation of completely processed transcripts. Immunoprecipitation studies documented the HIV-1 multiply spliced mRNAs encoding Tat, Rev, Nef are m7G-Capped transcripts, exhibit canonical CBC exchange to eIF4E, and experience eIF4E-dependent translation repressed by Torin-1 through hypo phosphorylated 4EBPs (Fig. 4A). By contrast, TMG-viral mRNAs are components of novel NCBP3/CBP80 translation mRNPs that are unaffected by mTOR inhibition [4]. Downregulation of NCBP3 by RNA interference was shown to abolish HIV-1 RNA specialized translation independently of eIF4E, and mimic the downregulation of RHA or TGS1 (Fig. 4B). In summary, TMG-Cap licenses biphasic HIV-1 translation in which synthesis of viral regulatory proteins is affected by mTOR, whereas specialized translation of virion structural and accessory proteinunaffected by mTOR sustains virion protein production after shut-off of regulatory protein synthesis.

#### PBS-segment is necessary for HIV-1 m7G-Cap hyper methylation that licenses specialized translation of virion proteins

A single A-to-C point mutation in the PBS-segment (A140C) disturbs the three-way junction structure recognized by RHA. Consequently, the mutant viral unspliced RNAs fail to undergo cap hypermethylation [4] (Fig. 4B). SAXS results identified the A140C mutant RNA adopted an extended hairpin structure compared to control (Fig. 4C) [3]. Results of isothermal caloriometry, gel shift analysis and reciprocal co-immunoprecipitation assays indicate that RHA interaction with PBS-segment of the HIV-1 Rev/RRE-dependent transcripts tethers TGS1 for cap hyper methylation [3, 4]. Recent in-cell probing of HIV-1 RNA secondary structures by Nanopore dimethylsulfate mutational profiling (Nano-DMS-MaP) reported that the HIV-1 unspliced RNA had different DMS reactivity than HIV-1 multiply spliced RNAs [78]. The structural differences in the PBS-segment provides a plausible explanation for why the multiply spliced RNAs fail to recruit RHA and TGS1 to undergo cap hyper methylation.

#### Consideration of specialized translation licensed by TMG-Cap/NCBP3-CBP80 and translation initiation by internal ribosome entry

HIV-1 sequences inclusive of PBS-segment were suggested to harbor an internal ribosome entry site (IRES) that promotes translation in an eIF4E-independent manner [84, 105]. Similar to specialized translation licensed by TMG-Cap, IRES-dependent translation is active during G2/M phase when eIF4E-dependent translation is repressed by hypo phosphorylated 4EBPs [105]. Mutagenesis identified that 5’UTR deletions or substitutions in PBS, DIS and 5’ splice site altered the 5’UTR IRES activity in bicistronic dual-luciferase reporter assays. While disruption of the stem loop upstream of PBS (nt 143–167) led to enhanced IRES activity, deletion of the loop residues downstream of PBS (nt 202–217), or mutations in DIS and 5’ splice site, were reported to negatively modulate IRES activity [84, 106]. It is worth noting that these mutant RNAs were likely to harbor misfolded PBS-segment, which results in deficient RHA binding, TGS1 loading, and ultimately the failure to undergo m7G-Cap hyper methylation.

In vitro translation of TMG-beta globin mRNAs in reticulocyte lysate has documented that TMG-Cap can support mRNA translation to protein, although the translation rate was reduced by a factor of 4 compared to m7G-Cap mRNAs [107]. This observed difference in translation rate aligns with the difference in de novo Gag synthesis rate between HIV-1 wildtype (WT) and mutant viruses carrying mutation in the 5’UTR disrupting PolyA [85]. The gag mRNA translation rates from alternative 5’UTRs in CD4 + T cells exhibited ~ 3-fold increase compared to the WT molecular clones that were licensed by TMG-capped mRNAs for specialized translation [4, 85]. These results offer a mechanistic explanation that the 5’UTR conformation is under positive selection for m7G-Cap hyper methylation; the lower basal translation rate by TMG-Cap-viral mRNAs molecular clones proved advantageous over the heightened translation rate exhibited by m7G-Cap-viral mRNAs [4, 104].

### Retroviruses have adaptively evolved sites susceptible for host RNA modification enzymes

PBS-segment is a substrate for epigenetic modification by hosts. The specific sites within the viral RNA and the stoichiometry of variously modified viral transcripts has been controversial. Moreover, investigations are warranted to define virological roles for internal modifications and potential conservation across the *Retroviridae*.

#### PBS-segment is a substrate for m6A methylation by host methyl transferase

The addition of a methyl group to the N6 position of an adenosine residue results in a modification known as N6-methyladenosine (m6A). The m6A modification of HIV-1 RNA has been shown to affect reverse transcription, mRNA stability, and viral protein expression [108, 109–111]. Recent study of the induction of type-I interferon by HIV-1 RNA in differentiated monocytes and primary macrophages identified that m6A-modified HIV-1 RNA may escape from host innate immune sensing by RIG-I [112].

The HIV-1 5’UTR contains two consensus sites for m6A modification: A198 in the PBS-segment and A242 in the DIS stem. Viral RNA fragments containing these modifications have been detected by sequencing of m6A-enriched mRNA from hosts (MeRIP-seq) [108, 109–111]. Results from HIV-1 transfected cells identified m6A modification in the 5’UTR and 3’UTR in preparations of intracellular RNA, whereas the m6A was solely identified in the 3’UTR of virion-associated RNA [113]. Study of ΔA_198_/ΔA_242_ mutant RNA indicated that Gag production increased and virion-associated transcripts decreased [113]. Such phenotype may be directly caused by m6A modification deficiency at these two positions, or by structural changes of the 5’UTR RNA resulting from the nucleotide deletions. Recent single-molecule transcriptomic analysis of full-length HIV-1 RNA in virions or infected cells by Oxford Nanopore direct RNA sequencing (DRS) identified several m6A sites, yet A198 and A242 are notably absent among them [114]. This absence may be due to the dynamic nature of m6A modification, which is regulated by viral RNA interactions with m6A writer, reader and eraser proteins. Consequently, some critical m6A sites may not consistently exhibit modification in all RNA transcripts or under all conditions. Further studies are warranted to determine whether m6A modification within the PBS-segment (A198) may be regulated by alteration in 5’UTR structures, viral or host protein interactions or the process of reverse transcription.

#### HIV-1 PBS-segment is a substrate for A-to-I editing by host adenosine deaminases that act on dsRNA (ADARs)

ADAR1 was reported to facilitate HIV-1 replication in human primary CD4 + T cells [91]. HIV-1 replication in peripheral blood mononuclear cells from ADAR1-deficient Aicardi-Goutières syndrome patients was restricted, and Gag protein production was ~ 50% decreased from the same amount of mRNA in ADAR1 down-regulated Jurkat cells [114]. HIV-1 virions produced from cells overexpressing enzymatically active ADAR1 were released more efficiently and exhibited enhanced infectivity [116]. Five adenosines in the HIV-1 5’UTR were found to undergo A-to-I editing. Four of these adenosines are located in the residues upstream of PBS-segment (in PolyA) and one is located in PBS-segment [116]. Further studies are warranted to elaborate potential inferences in post-transcriptional expression, reverse transcription, or host innate sensing of viral or virion RNA attributable to A-to-I editing.

## Part 4. Summary of retroviral PBS-segment issues and perspectives on future scope

Historically, tRNA annealing that primes reverse transcription initiation was the first function identified for the PBS-segment. It has been well-established that tRNA annealing to vRNA results in cDNA elongation that copies the U5 and R sequences into -sssDNA. These vRNA residues are highly conserved and after the completion of reverse transcription, the U5 residues encompass ~ 12 base pairs required for integration of the cDNA product into the host chromosome (*att* site) (Fig. 3). Mutational analysis of the *att* site has been carried out by in vitro assays [117] and cell-based molecular genetics assays [118] that have demonstrated A-rich bulge structure and the CA dinucleotide of PBS-segment are recognized by the IN protein. Mutations in this region can be detrimental for both cDNA integration and reverse transcription, thus emphasizing the overlapping information content within the PBS-segment. As discussed in Part 2, mutations made to study one aspect of PBS-segment structure or function may have unintended consequences on other replication events, thus confounding straightforward data interpretation.

In addition to the *att* site, the PBS-segment also contains a conserved GAmotif (Fig. 3) that participates in the balanced expression of viral unspliced and spliced transcripts [95]. While the GAmotifs may be recognized as a primary sequence motif, their function may be subject to perturbation by the dynamic structure of the 5’UTR (Fig. 2). As discussed in Part 2, structural dynamics of the 5’UTR can be modulated by several biologically important variables including hyper methylation of m7G-Cap to TMG-Cap and heterogeneous TSS, both of which may alter G: C pairings within the 5’UTR and the dynamic interaction with cognate RNA-binding proteins necessary for infectious virion production.

Featured by the relative scarcity of canonical base pairs, PBS-segment likely represents one of the most dynamic and least-tightly paired regions in the highly structured 5’UTR, lending flexibility to shapeshift for the many intertwined RNA transactions that coordinate events in retroviral replication. Further studies are crucial to fully comprehend how the PBS-segment dynamics manifest interactions as diverse as vRNA with cognate tRNA; host RNA helicase and the RTIC; cDNA engaging with IN tethered to chromatin for completion of early replication steps. PBS-segment dynamics selective to nascent Rev/RRE-dependent transcripts subvert the process of CBC exchange and shift HIV-1 translation initiation from the canonical m7G-Cap/eIF4E pathway to the specialized translation pathway licensed by TMG-Cap/NCBP3-NCBP1. Prior to the identification of TMG-Cap-NCBP3 interaction in specialized translation, Snurportin had been the only known TMG-Cap binding protein [119]. Historically, studies of retroviral RNAs continually have illuminated specialized pathways in host RNA biology. Though future studies are warranted, consensus has been reached that PBS-segment is a key HIV-1 motif, modulating vRNA reverse transcription and cDNA integration and, by perturbing canonical host events at the m7G-Cap, for specialized translation licensed by TMG-Cap on select viral RNAs.

## Data Availability

No datasets were generated or analysed during the current study.
